# 944. Triaging Wound Care in a Burn Center: Enhancing Communication

**DOI:** 10.1093/jbcr/irag033.511

**Published:** 2026-04-06

**Authors:** Carson Bair, Ashley Rowe, Chloe E Chapin-Grabowski, Nicolas Tran, Sai Ram Velamuri

**Affiliations:** Tampa General Hospital Regional Burn Center, Tampa, FL; Tampa General Hospital Regional Burn Center, Tampa, FL; Tampa General Hospital, Tampa, FL; Tampa General Hospital Regional Burn Center, Tampa, FL; University of South Florida Health Morsani College of Medicine, Tampa, FL

## Abstract

**Introduction:**

Interdisciplinary communication is crucial for safe and effective wound care for burn patients. At a verified burn center, a triage protocol was implemented to streamline communication and guide treatment based on infection risk, ranging from Level 1 for non-infected wounds to Level 4 for risk of life-threatening sepsis. This study assessed the impact of the protocol on communication and confidence in clinical decision making via pre- and post- surveys.

**Methods:**

All nurses and providers at the burn center attended at least 1 education session before protocol implementation. Pre-surveys were administered following these sessions. Post-surveys were administered after 5 months of protocol implementation. All surveys were optional and tailored to varying disciplines (i.e., one for nurses and another shared by physicians and advance practice providers). Surveys assessed protocol perceptions, changes in interdisciplinary communication, and confidence in clinical decision-making. Mann–Whitney U Tests evaluated differences in pre- and post-survey responses, with *p*<.05 indicating statistical significance.

**Results:**

Twenty-five providers and 18 nurses (17 in the pre-survey) participated. Familiarity with the triage levels and respective care protocols increased significantly for nurses and providers. Providers also reported improved perceived effectiveness of care with the protocol and less frequent communication issues. Providers and nurses reported increased confidence in clinical decision making but not statistically significant. Nurses consistently reported high levels of understanding of clinical orders.

**Conclusions:**

Survey responses demonstrated notable improvements in interdisciplinary communication and perceived effectiveness of care. These findings suggest the implementation of the burn triage protocol successfully increased staff communication and adherence to care guidelines. Future research should look at patient outcomes to determine impact of the protocol on infection and complication rates.

**Applicability of Research to Practice:**

Implementing a triage protocol for wound care can improve interdisciplinary communication and the quality of burn care to ultimately improve long-term patient outcomes. Structured protocols are an effective method to enhance workflow while supporting burn care consistent with evidence-based guidelines.

**Funding for the study:**

N/A.

